# Pro-106-Ser mutation and EPSPS overexpression acting together simultaneously in glyphosate-resistant goosegrass (*Eleusine indica*)

**DOI:** 10.1038/s41598-017-06772-1

**Published:** 2017-07-27

**Authors:** Javid Gherekhloo, Pablo T. Fernández-Moreno, Ricardo Alcántara-de la Cruz, Eduardo Sánchez-González, Hugo E. Cruz-Hipolito, José A. Domínguez-Valenzuela, Rafael De Prado

**Affiliations:** 1 Department of Agronomy, Gorgan University of Agricultural Sciences and Natural Resources, 49189-43464 Gorgan, Iran; 2 Department of Agricultural Chemistry and Edaphology, Campus of Rabanales, University of Cordoba, 14071 Cordoba, Spain; 3 Departamento de Entomologia/BIOAGRO, Universidade Federal de Viçosa, 36570-900 Viçosa, Brazil; 4 Department of Agricultural Parasitology, Chapingo Autonomous University, 56230 Texcoco, Mexico; 5 Bayer CropScience Mexico, 11520 Mexico City, Mexico

## Abstract

Glyphosate has been used for more than 15 years for weed management in citrus groves in the Gulf of Mexico, at up to 3–4 applications per year. Goosegrass (*Eleusine indica* (L.) Gaertn.) control has sometimes failed. In this research, the mechanisms governing three goosegrass biotypes (Ein-Or from an orange grove, and Ein-Pl1 and Ein-Pl2 from Persian lime groves) with suspected resistance to glyphosate were characterized and compared to a susceptible biotype (Ein-S). Dose-response and shikimate accumulation assays confirmed resistance of the resistant (R) biotypes. There were no differences in glyphosate absorption, but the R biotypes retained up to 62–78% of the herbicide in the treated leaf at 96 h after treatment (HAT), in comparison to the Ein-S biotype (36%). The 5-enolpyruvylshikimate-3-phosphate synthase (EPSPS) activity in the Ein-Or and Ein-S biotypes was over 100-fold lower than the Ein-Pl1 and Ein-Pl2 ones. The latter showed a high EPSPS-basal activity, a mutation at Pro-106-Ser position in the EPSPS gene, and EPSPS overexpression. The EPSPS basal and EPSPS overexpression were positively correlated. The R goosegrass biotypes displayed poor glyphosate translocation. Furthermore, this grassweed showed, for the first time, two mechanisms at the target-site level (Pro-106-Ser mutation + EPSPS overexpression) acting together simultaneously against glyphosate.

## Introduction

Goosegrass (*Eleusine indica* (L.) Gaertn.), an annual-C_4_ summer grass plant, is one of the world’s worst weeds. It comes from Africa and is widely spread over tropical, subtropical regions and temperate regions of the world^1, 2^. Goosegrass is a problematic weed mainly on lawns, in annual crops and in fruit orchards^3, 4^. In Mexico, goosegrass is widespread in 25 states^5^. Depending on the crop, different herbicides have been recommended for goosegrass control^6^. However, herbicide use as the primary weed management approach has resulted in goosegrass resistance to different herbicide groups, such as acetohydroxyacid synthase (ALS) inhibitors, acetyl-CoA carboxylase (ACCase) inhibitors, photosystem I and II (PSI and PSII) inhibitors, microtubule inhibitor, glutamine synthetase and 5-enolpyruvylshikimate-3-phosphate synthase (EPSPS; EC 2.5.1.19) inhibitors^7^. Even one biotype from Malaysia showed multiple resistance to three non-selective herbicides, glufosinate, glyphosate and paraquat^8^.

Glyphosate (N-phosphonomethyl-glycine) is a post-emergence, non-selective and systemic herbicide that is used extensively to control a wide spectrum of weeds in orchards, soil conservation systems and non-cropped (fallow) areas^9^. Glyphosate inhibits EPSPS, disrupting the biosynthesis of phenylalanine, tyrosine, tryptophan and others secondary aromatic products^10^. This is due to the complex effects caused by glyphosate on the shikimate pathway, the photosynthetic processes and the oxidative events of plants^11^, triggering the expression of other chloroplast enzymes^12^. Recently, the expression of the phosphofructokinase (PFK: EC 2.7.1.11) enzyme has been strongly associated with EPSPS expression^13^.

The widespread reliance on glyphosate for weed control in recent decades, and the introduction of glyphosate-resistant crops has resulted in a large number of weeds becoming resistant to this herbicide^7, 14^. Glyphosate resistance mechanisms can be categorized into two classes: target site resistance (TSR) and non-target site resistance (NTSR)^15^. TSR mechanisms are a result of mutations during encoding of the EPSPS gene^1, 4, 13, 15, 16^, overexpression of the EPSPS enzyme^4, 13, 17^, and/or enhanced basal EPSPS activity^17, 18^. NTSR mechanisms are due to reduced absorption and translocation^18–21^, vacuolar sequestration^22^, and metabolism of the herbicide into non-toxic compounds^23^.

The state of Veracruz, on the Gulf of Mexico, is the main citrus fruit producing region in Mexico, with Persian lime being the most important crop because of its large production and its economic value^24^. In addition to mowing, few other approaches or herbicides are employed to manage weeds in citrus production systems (Dominguez-Valenzuela, personal communication) other than glyphosate^25^. To date, *Bidens pilosa* ^16^ and *Leptochloa virgata* ^17^ have been identified as resistant to this herbicide in citrus groves in the states of Veracruz and Puebla. However, weed control with glyphosate has also failed for other weeds in the citrus production systems of this region.

The objectives of this study were as follows: 1) to confirm resistance of goosegrass to glyphosate; and 2) to study the resistance mechanisms involved in the resistant biotypes collected from citrus fruit groves in Mexico.

## Results

### Dose-response

**Figure 1:**
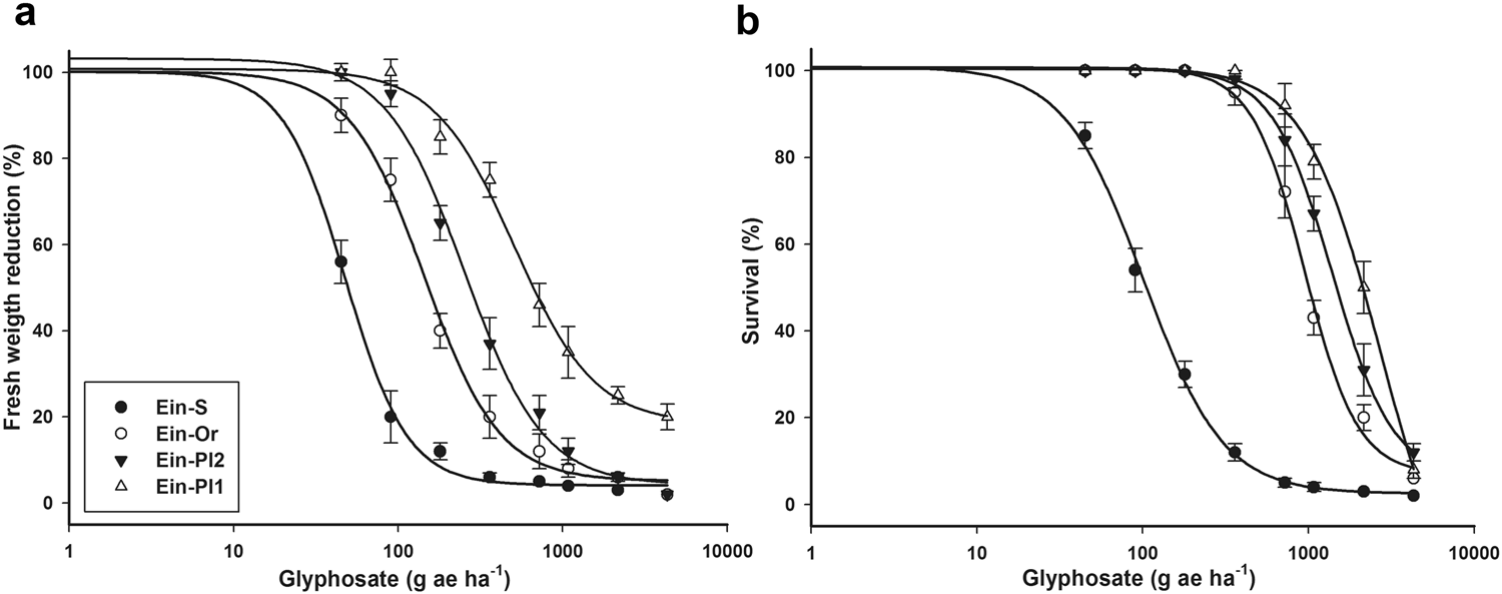
Log–logistic curves of glyphosate -susceptible and -resistant goosegrass (*Eleusine indica*) biotypes collected in citrus groves in Veracruz, Mexico, evaluated at 21 DAT. (**a**) Dose-response curve with respect to percentage of fresh mass reduction. (**b**) Dose-response curve with respect to percentage of survival. Vertical bars represent the standard error of the mean (*n* = 10).

The survival and fresh weight of the four goosegrass biotypes decreased with increasing glyphosate doses (Fig. 1). The three suspected resistant (R) biotypes collected from the citrus-growing region of Veracruz, Mexico [Ein-Pl1 and Ein-Pl2 biotypes from Persian lime (*Citrus latifolia* Tan.) groves and Ein-Or biotype from an orange (*Citrus sinensis* Osbeck) grove]; presented resistance to glyphosate. Therefore, the glyphosate doses used to reduce the fresh weight (GR_50_) and to kill a plant population (LD_50_) by 50% of the R biotypes ranged from 136 to 556, and from 974 to 1938 g per ha, respectively. According to these parameters, the resistance indexes (RI) ranged between 2.6 and 15.9. LD_90_, and GR_90_ corroborated the resistance of these biotypes (Table 1).

**Table 1:** Parameters of the log-logistic equation^a^ used to calculate the glyphosate dose (g ae per ha) required to reduce the fresh weight (GR) and survival percentage (LD) by 50 or 90% of the glyphosate-resistant and -susceptible goosegrass (*Eleusine indica*) biotypes collected in citrus groves from Veracruz, Mexico.

**Fresh weight reduction (GR)**											
**Biotypes**	***c***	***d***	***b***	**GR**_**50**_	**CI95%** ^**b**^	**RI** ^**c**^	***P***	**GR**_**90**_	**CI95%** ^**b**^	**RI** ^**c**^	***P***
Ein-Pl1	2.34	101.90	1.54	555.6	46.6	10.4	0.0001	2297.4	267.8	20.5	0.0038
Ein-Pl2	7.17	102.07	1.97	267.1	24.9	5.0	0.0001	811.0	147.3	7.2	0.0001
Ein-Or	2.92	102.49	2.04	137.5	13.4	2.6	0.0001	418.9	72.0	3.7	0.0006
Ein-S	0.93	100.65	2.91	53.4	7.6	—	—	111.9	15.8	—	—
**Survival percentage (LD)**											
**Biotypes**	***c***	***d***	***b***	**LD**_**50**_	**CI95%** ^**b**^	**RI** ^**c**^	***P***	**LD**_**90**_	**CI95%** ^**b**^	**RI** ^**c**^	***P***
Ein-Pl1	0.12	99.45	3.69	1938.3	366.1	15.9	0.0001	3956.0	521.4	18.9	0.0001
Ein-Pl2	0.62	100.08	4.96	1442.6	244.3	11.9	0.0001	2244.5	396.0	10.7	0.0001
Ein-Or	2.72	100.15	2.99	973.8	184.3	8.0	0.0001	2028.7	337.6	9.7	0.0001
Ein-S	0.57	100.40	2.89	121.7	23.7	—	—	209.2	33.8	—	—

^a^ *Y* = *c* + {(*d* − *c*)/[1 + (*x*/*g*)^*b*^]} where; *Y* is the percentage of fresh weight and/or survival with respect to the control, *c* and *d* are the the lower and upper asymptotes, *b* is the slope of the curve at the inflection point, *g* the herbicide dose at the inflection point (i.e. GR_50_ or LD_50_), and *x* (independent variable) is the glyphosate dose. ^b^CI values are the upper and lower limits (±) of the 95% confidence intervals (*n = *10). ^c^RI = Resistance index (R/S) calculated using the corresponding ED_50_, or LD_50_ values of the resistant biotype with respect to the susceptible one.

### Shikimic acid accumulation

**Figure 2:**
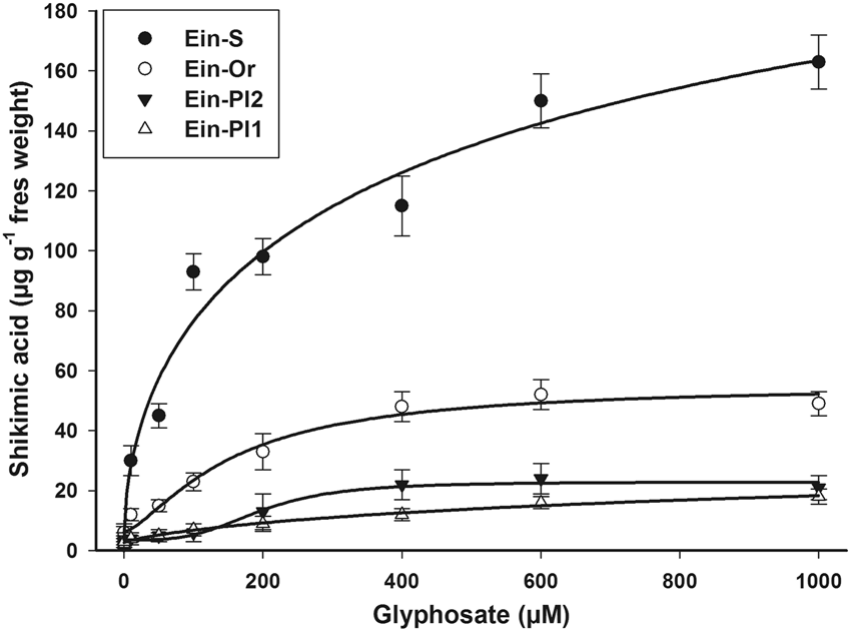
Shikimic acid accumulation of the glyphosate -susceptible and -resistant goosegrass (*Eleusine indica*) biotypes collected in citrus groves in Veracruz, Mexico, at different glyphosate concentrations. Vertical bars represent the standard error of the mean (*n* = 3).

Both the R and S biotypes of goosegrass accumulated shikimic acid as the glyphosate concentrations increased. The greater accumulation of shikimic acid (approximately 160 µg g^−1^ fresh weight) was exhibited by the Ein-S biotype. The R biotypes accumulated between 3.3 and 8.1 times less shikimic acid at 1000 µM glyphosate, with the lowest amounts accumulated by the Ein-Pl1 and Ein-Pl2 biotypes (Fig. 2).

### Absorption and translocation

**Figure 3:**
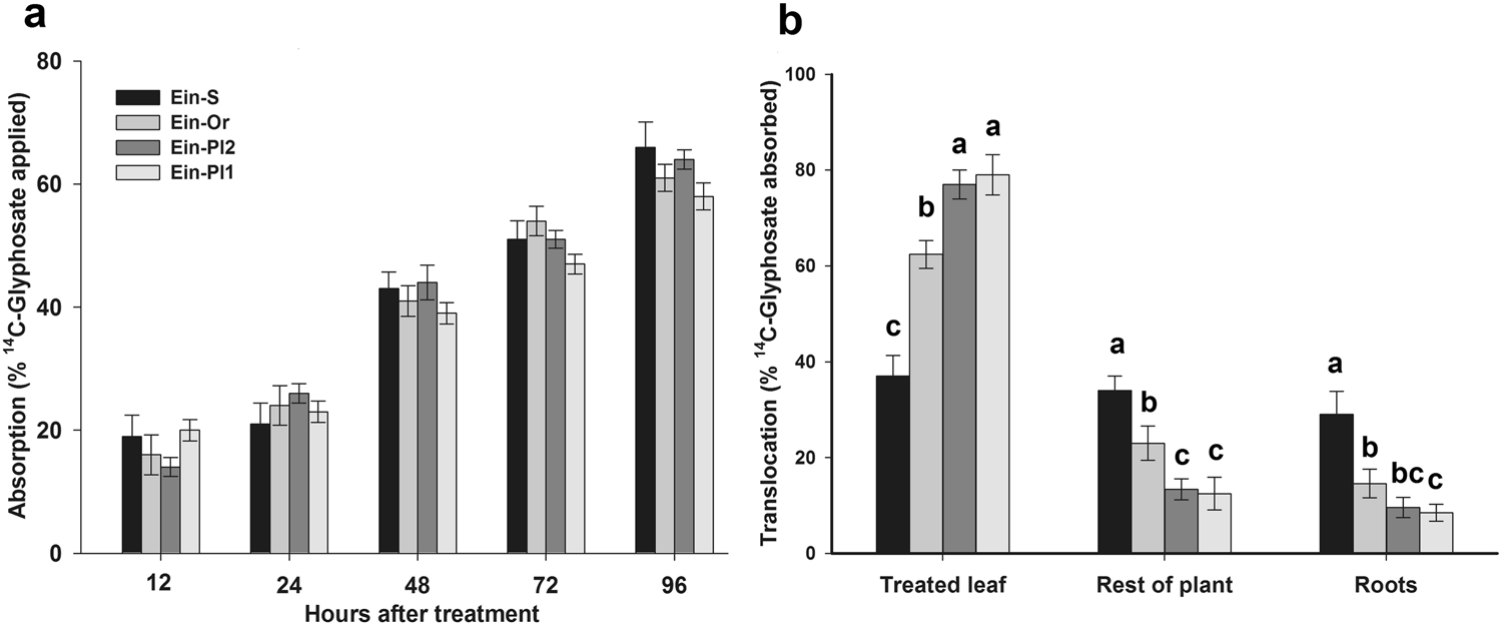
^14^C-Glyphosate absorption and translocation in glyphosate-susceptible and -resistant plants of goosegrass (*Eleusine indica*) biotypes collected in citrus groves in Veracruz, Mexico. (**a**) ^14^C-glyphosate absorption in goosegrass plants from 12 to 96 HAT. (**b**) ^14^C-glyphosate translocation in goosegrass plants at 96 HAT. Different letters are significantly different at 95% probability determined by Tukey’s test. The vertical bars represent the standard error of the mean (*n* = 5).

Foliar ^14^C-glyphosate absorption showed no differences between goosegrass biotypes. At 12 h after treatment (HAT), the ^14^C-glyphosate absorption rate ranged from 14.3 to 20.1%, and from 58.4 to 66.2% at 96 HAT (Fig. 3).

However, the ^14^C-glyphosate translocation exhibited differences between 12 to 96 HAT. The Ein-S biotype presented the highest translocation rate at 96 HAT, moving most of the absorbed glyphosate from the treated leaf to other plant parts (33.8 and 29.3% to the rest of the plant and roots, respectively). Conversely, the R biotypes retained a larger amount of herbicide in the treated leaf, with 62.4, 76.3 and 79.6% of the glyphosate absorbed by the Ein-Or, Ein-Pl2 and Ein-Pl1 biotypes, respectively, evaluated simultaneously. The R biotype Ein-Or translocated slightly more glyphosate to the rest of the plant and roots than the others two R biotypes (Fig. 3).

### Glyphosate metabolism

More than 90% of the applied glyphosate was not metabolized by the goosegrass plants at 4 DAT. At this time, small amounts of AMPA (aminomethyl phosphonate) (36.−6.6%) and glyoxylate (1.6–2.4%) were detected in all goosegrass biotypes. A similar amount of metabolized glyphosate in the R and S biotypes, as well as the non-detection of sarcosine, suggested that metabolism is not a resistance mechanism (Table 2).

**Table 2:** Glyphosate metabolism expressed as percentage of total glyphosate and their metabolites in the glyphosate-resistant and -susceptible goosegrass (*Eleusine indica*) biotypes collected in citrus groves from Veracruz, Mexico.

Biotypes	glyphosate	AMPA	glyoxylate	sarcosine
Ein-Pl1	93.2 ± 2.6	4.6 ± 1.4	2.1 ± 0.6	ND
Ein-Pl2	91.7 ± 2.3	6.6 ± 2.2	1.7 ± 1.1	ND
Ein-Or	92.8 ± 5.9	4.8 ± 1.8	2.4 ± 1.2	ND
Ein-S	95.3 ± 4.1	3.6 ± 0.9	1.6 ± 0.4	ND
*P*	0.3765	0.1395	0.0672	ND

Values represent mean (*n* = 6). ±Standard error. *AMPA* aminomethyl phosphonate. *ND* not detected.

### EPSPS enzyme activity

**Figure 4:**
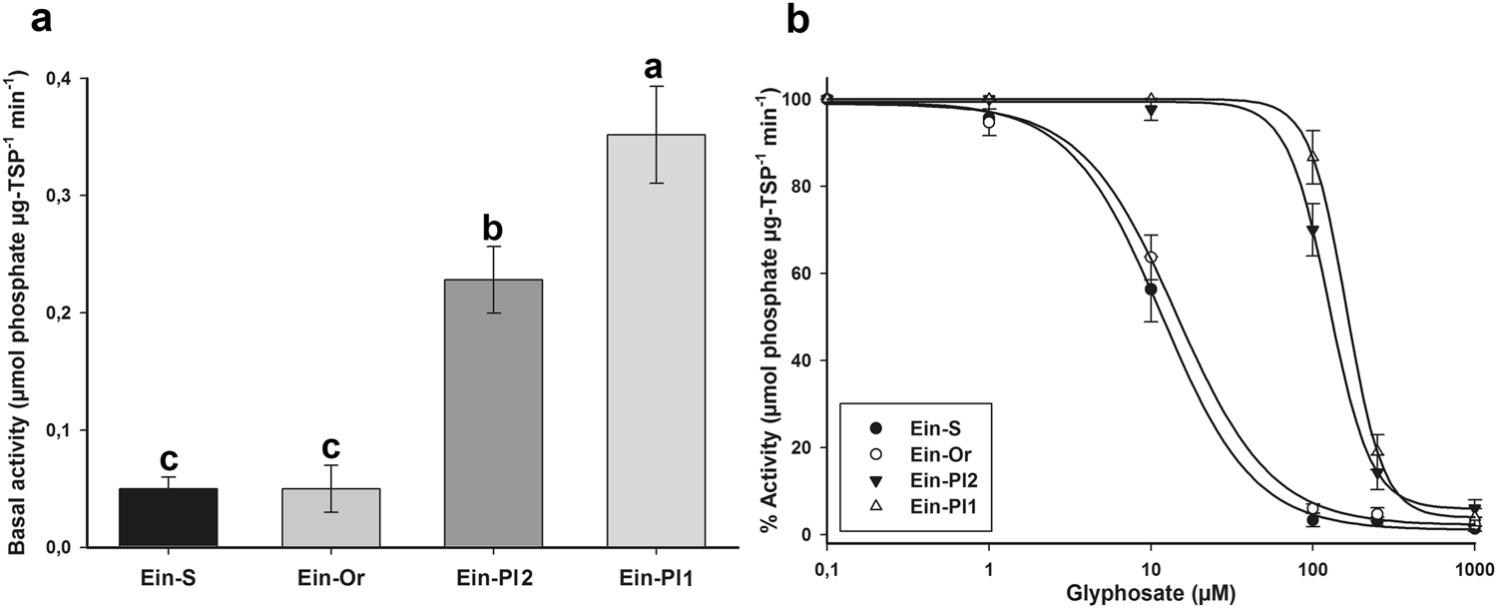
EPSPS activity in glyphosate-susceptible and -resistant plants of goosegrass (*Eleusine indica*) biotypes collected in citrus groves in Veracruz, Mexico. (**a**) Basal EPSPS activity in glyphosate -susceptible and -resistant goosegrass plants. Histograms represent the treatment means and vertical bars ± standard error (*n* = 3). (**b**) EPSPS enzyme activity expressed as a percentage of the untreated control in leaf extracts of plants from glyphosate-susceptible and resistant goosegrass biotypes. Vertical bars represent ± standard error (*n* = 3).

Significant differences were found in the basal EPSPS activity. The Ein-Pl1 biotype presented the highest basal activity (0.35 μmol of phosphate released per μg total soluble protein (TSP)^−1^ min^−1^), whereas the Ein-Pl2, Ein-Or and Ein-S biotype averages were 0.20, 0.03 and 0.04 of μmol μg TPS^−1^ min^−1^, respectively (Fig. 4).

The EPSPS enzyme was inhibited by glyphosate in the S and R biotypes. The glyphosate concentration that inhibited EPSPS activity by 50% (I_50_) in the Ein-S biotype was 2.8 µM. With respect to this parameter, the RIs of Ein-Or, Ein-Pl2 and Ein-Pl1 biotypes were 1.2, 10.8 and 13.8, respectively, compared to the S biotype. There were no differences in the EPSPS activity between the Ein-Or and Ein-S biotypes (Table 3).

**Table 3:** Parameters of the log-logistic equationa used to calculate the glyphosate concentration dose (µM) required to reduce the EPSPS activity reduction (I_50_) by 50% of the glyphosate-resistant and -susceptible goosegrass (*Eleusine indica*) biotypes collected in citrus groves from Veracruz, Mexico.

Biotypes	*c*	*d*	*b*	I_50_	CI95%^b^	RI^c^	*P*
Ein-Pl1	3.91	100.00	3.82	161.1	18.8	13.8	<0.0001
Ein-Pl2	5.90	99.42	3.37	125.9	14.6	10.8	<0.0001
Ein-Or	2.14	98.93	1.48	14.4	2.8	1.2	<0.0001
Ein-S	1.07	99.37	1.53	11.7	3.3	—	<0.0001

^a^ *Y* = *c* + {(*d* − *c*)/[1 + (*x*/*g*)^*b*^]} where; *Y* is the percentage of EPSPS activity with respect to the control, *c* and *d* are the lower and upper asymptotes, *b* is the slope of the curve at the inflection point, I_50_ the herbicide dose at the inflection point, and *x* (independent variable) is the glyphosate concentration. ^b^CI values are the upper and lower limits (±) of the 95% confidence intervals (*n* = 3). ^c^RI = Resistance index = I_50_R/I_50_S.

### EPSPS gene sequence

The amplified fragment of the EPSPS gene included Thr-102 and Pro-106 positions, the point mutations conferring glyphosate resistance, presenting a homology of above 98% between goosegrass biotypes. The obtained EPSPS sequences were aligned with the GenBank accessions (R-J417033 and S-J417034) of goosegrass that were reported by Baerson *et al*.^1^. In the Thr-102 position, no mutation was found. At the Pro-106 position, the Ein-Pl1 and Ein-Pl2 biotypes exhibited a codon change from CCA to TCA, resulting in an amino acid substitution from Proline to Serine (Table 4).

**Table 4:** Partial alignment of nucleotides of EPSPS gene of the glyphosate-resistant and -susceptible goosegrass (*Eleusine indica*) biotypes collected in citrus groves from Veracruz, Mexico.

Position^a^	99	100	101	102	103	104	105	106	107
*Accession* ^b^									
R-J417033	AAT	GCT	GCA	ACT	GCA	ATG	CGA	**TCA** ^c^	TTG
S-J417034	AAT	GCT	GCA	ACT	GCA	ATG	CGA	CCA	TTG
*Biotype*									
Ein-Pl1	AAT	GCT	GCA	ACT	GCA	ATG	CGA	**TCA**	TTG
Ein-Pl2	AAT	GCT	GCA	ACT	GCA	ATG	CGA	**TCA**	TTG
Ein-Or	AAT	GCT	GCA	ACT	GCA	ATG	CGA	CCA	TTG
Ein-S	AAT	GCT	GCA	ACT	GCA	ATG	CGA	CCA	TTG

^a^Amino acid position based on the start codon (ATG) of *Arabidopsis thaliana* (GenBank: CAA29828.1) EPSPS sequence. ^b^Genbank accession reported by Baerson *et al*.^1^. ^c^Codon change from CCA to TCA resulting in an amino acid substitution from Proline to Serine that confers resistance to glyphosate.

### EPSPS gene expression

**Figure 5:**
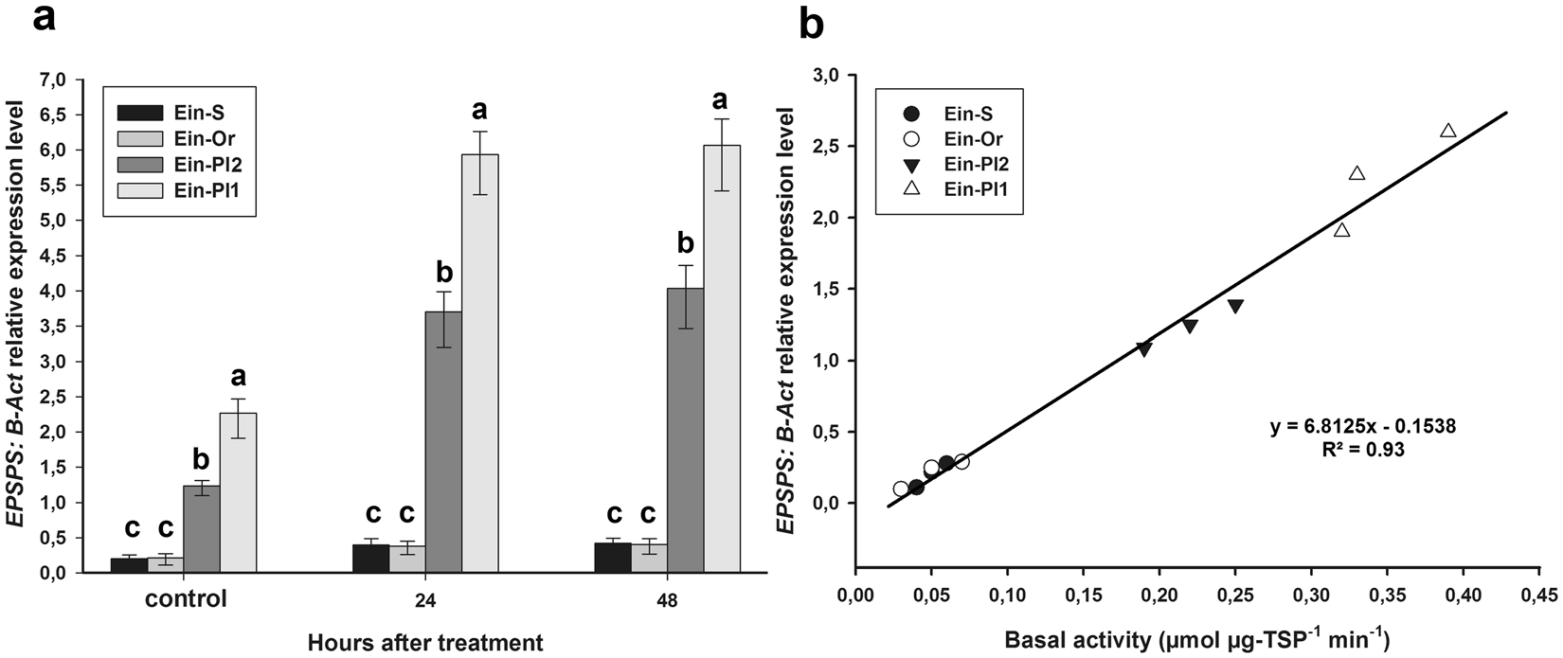
EPSPS gene expression relative to β-Actin for glyphosate -susceptible and -resistant plants of goosegrass (*Eleusine indica*) biotypes. (**a**) EPSPS expression level for glyphosate-susceptible and -resistant goosegrass plants before and after glyphosate treatment. Histograms represent the treatment means and vertical bars ± standard error (*n* = 6). (**b**) correlation between the EPSPS expression (control) and EPSPS basal activity.

The EPSPS gene expression quantified from cDNA in the goosegrass biotypes was determined before and after glyphosate application. EPSPS gene expression relative to the *β*-Actin gene before treatment (control) ranged from 0.11 to 0.28, 0.10 to 0.25, 1.09 to 1.36 and 1.90 to 2.58 for the Ein-S, Ein-Or, Ein-Pl2 and Ein-Pl1 biotypes, respectively. After glyphosate treatment, the EPSPS expression level ranged between 0.23 to 0.54 for the Ein-S and Ein-Or biotypes, and 5.18–6.73 and 3.22–4.48 for the Ein-P1 and Ein-Pl2 biotypes, respectively. Furthermore, the EPSPS expression level of the control was positively correlated with the EPSPS basal activity (Fig. 5).

## Discussion

### Determining resistance

Glyphosate resistance was confirmed in three goosegrass biotypes, even in the Ein-Or biotype from the orange grove, which had the lowest GR_50_ values, because the glyphosate field dose (720 g per ha) used in citrus groves from Veracruz was not enough to achieve full control, because this biotype presented a LD_50_ of 973.8 g per ha. This is consistent with the fact that producers care more about weed management in Persian lime groves than in orange groves due to the greater economic profitability of limes, which induces more frequent glyphosate use in these groves. Outside the flowering and fruit set seasons, eventually 2,4-D is applied in the mixture with lower glyphosate doses to improve the control of broadleaf weeds (Domínguez-Valenzuela, personal communication). However, this practice has not reduced the selection pressure exerted by glyphosate and, unfortunately, these results confirm a third case of glyphosate resistance in the citrus region of the Gulf of Mexico, after identification of *L. virgata* in 2010^25^ and *B. pilosa* in 2014^16^. The RIs estimated based on GR_50_ and LD_50_ parameters, are in agreement with the RIs estimated for other biotypes/populations of goosegrass^2–4, 26–28^.

The amount of shikimic acid accumulated by goosegrass R biotypes indicated that the selection pressure exerted by glyphosate in these biotypes causes scant sensitivity to this herbicide. Differential accumulation of shikimic acid occurs when glyphosate does not reach the target-site in sufficient amounts, inhibiting EPSPS in different proportions^17^. For glyphosate to be lethal in plants with a low shikimic acid accumulation, larger amounts of herbicide are required^16^. The different shikimic acid accumulation patterns between R and S goosegrass biotypes were similar to those observed in the dose-response assays, indicated high glyphosate susceptibility of the S biotype and a different resistance level of R biotypes. This suggests that the mechanisms of resistance involved in the R biotypes are different from each other. The shikimic acid accumulation has been widely reported as discriminating resistance to glyphosate^4, 23, 29–31^.

### Exploring the NTSR mechanism involved

The absorption of glyphosate is influenced by leaf morphological and physiological traits^32^, which has been suspected to occur through active transporters and by passive diffusion down a concentration gradient in cells^12^. Goosegrass biotypes presented similar ^14^C-glyphosate absorption patterns over time after application. This suggests that the R and S goosegrass biotype leaf traits have not been altered by glyphosate selection pressure, thereby allowing absorption in the same proportion. Therefore, absorption is not implicated in the resistance to glyphosate of R goosegrass biotypes. This mechanism is not usually involved in glyphosate resistance, and only some grassweed species, such as *Digitaria insularis* ^23^, *L. virgata* ^18^, *Lolium multiflorum* ^20^ and *Sorghum halepense* ^21^, have registered differences in their absorption patterns.

Glyphosate is a systemic herbicide and must reach the enzymatic sites of root and shoot meristems in order to act^10^. Any reduction in its translocation to these active and sensitive sites has a negative effect on its efficiency^33^. R goosegrass biotypes (Ein-Or, Ein-Pl1 and Ein-Pl2) exhibited less glyphosate translocation to the rest of the plant and roots than the S biotype (Ein-S), retaining it mainly within the treated leaves. The exact mechanism that reduces glyphosate translocation is not fully known, but a possible change in the plasma membrane transporters that restricts the glyphosate movement inside of resistant plants^19^ has been considered. It has also been suggested that there is an unknown barrier altering the subcellular distribution of glyphosate and keeping it away from the target site^34^. This barrier may exist either in the phloem system or in the mesophyll cells, where glyphosate has to enter to be translocated^35^. The Ein-Or biotype was selected by glyphosate less often than the Ein-Pl1 and Ein-Pl2 biotypes. This explains why its translocation rate is higher with respect to the latter biotypes but was significantly lower than the Ein-S biotype. Therefore, differential translocation of the herbicide can be considered one of the glyphosate resistant mechanisms in these R goosegrass biotypes. Similar restrictions in glyphosate translocation were reported for different grassweed species, such as the following: *D. insularis* ^23^ *, Lolium multiflorum* ^36^ *, L. perenne* ^37^, *L. rigidum* ^30^ *, Leptochloa virgata* ^18^, *S. halepense* ^21^, among others.

On the other hand, glyphosate metabolism has been verified as not involved in the resistance to glyphosate in R goosegrass biotypes. Degradation of glyphosate has been documented in a few weed species, such as the following: *D. insularis* ^23^ and *Parthenium hyterophorus* ^38^. Although metabolism could influence resistance to glyphosate, there is no evidence that it plays an important role in it^39^, and it has been rarely studied or reported as endowing resistance to glyphosate^15^. Our results were consistent with other studies in *B. pilosa* ^16^, *C. canadensis* ^40^ *, Echinoclhoa colona* ^17^ and *L. virgata* ^18^, which demonstrated that this mechanism does not contribute to glyphosate resistance.

### Exploring the TSR mechanism involved

The goosegrass biotypes of Ein-S and Ein-Or presented similar basal EPSPS levels and glyphosate response patterns. This indicates that the R biotype Ein-Or is susceptible to glyphosate at a target-site level. The EPSPS enzyme of glyphosate R plants could be sensitive to glyphosate when the EPSPS gene had no mutations^18, 41^. This explains why the Ein-Or biotype had similar susceptibility to glyphosate as that of the Ein-S biotype, suggesting that this R biotype does not employ target-site mechanisms against this herbicide. On the other hand, the Ein-Pl1 and Ein-Pl2 biotypes presented high basal EPSPS activity, and larger amounts of glyphosate were required to inhibit EPSPS activity by 50%. Basal activity differences are associated with a greater EPSPS gene amplification, and consequently, the overexpression of its enzyme^42^. This implies that, in addition to reduced translocation, Ein-Pl1 and Ein-Pl2 biotypes presented at least one target-site mechanism against glyphosate. Different R goosegrass populations from China^4, 13^, and other glyphosate resistant grassweed species have displayed high levels of basal EPSPS activity compared to their respective susceptible ones^17, 42^, and higher glyphosate concentrations were necessary to inhibit the EPSPS activity by 50%.

EPSPS gene sequencing demonstrated that the Ein-Pl1 and Ein-Pl2 biotypes possessed a mutation at Pro-106 position. Amino acid substitution in this genomic EPSPS position from Proline to Serine, Alanine, Threonine and/or Leucine, has been widely reported in mono- and dicotyledonous weeds, endowing partial resistance to glyphosate, in some cases accompanied by a NTSR mechanism^15^. Some grassweed species, which have shown a mutation in combination with other mechanism (TSR + NTSR) in the last two years, are *C. virgata* ^31^ *, E. colona* ^17^ *, L. virgata* ^18^, *L. rigidum* ^30^ and *Poa annua* ^29^. Recently, the double mutation commonly known as TIPS (Thr-102-Ile + Pro-106-Ser), has been documented in glyphosate resistant populations of goosegrass^4, 26^, and *B. pilosa* ^16^, endowing them with a high degree of resistance.

EPSPS expression in cDNA was differed in the R goosegrass plants of the Ein-Pl1 and Ein-Pl2 biotypes with respect to plants of the Ein-S and Ein-Or biotypes. The EPSPS expression of the R and S biotypes before glyphosate treatment (control) was strongly and positively correlated with their EPSPS basal activity, indicating that the EPSPS expression was proportionate to the amount of transcribed EPSPS enzyme. There is a positive correlation between the EPSPS gene copy number and the EPSPS transcription^43^. It is possible that the goosegrass biotypes have different EPSPS genomic copy numbers from each other. However, the EPSPS gene copy numbers were not studied in the goosegrass biotypes, because the manifestation of more EPSPS gene copies does not always result in higher protein transcription^44^. Furthermore, an increase in EPSPS expression was observed after treatment in response to the stress exerted by glyphosate. This level of expression remained constant at 24 and 48 HAT. Similar results were observed in R goosegrass populations from China^4^, where the EPSPS expression levels from 6 to 72 HAT treated at 922.5 g per ha were evaluated and remained constant after application. The EPSPS expression level depends more on the dose of glyphosate applied than the time period evaluated. Goosegrass (R and S) plants exhibited a gradual increase in the EPSPS expression with increased concentrations of glyphosate^2^. Some resistant grassweed species presenting EPSPS copy numbers and/or overexpression as glyphosate resistance mechanisms are as follows: *B. diandrus* ^45^ *, Kochia scoparia* ^44^ and *L. perenne* ^42, 46^. These results indicate that the Ein-Pl1 and Ein-Pl2 goosegrass biotypes from Persian lime groves have evolved a mutation and overexpression of the target site, which gives them greater resistance to glyphosate due to the different selection pressure exerted by this herbicide in these groves compared to the orange groves.

It is important to note that, so far, known cases of glyphosate resistance involving the target-site were only due to point mutations (the single Pro-106 mutation^1, 4, 16–18, 26, 29–31^, or the double Thr-102 + Pro-106 one^4, 16, 26^) or to EPSPS amplification^2, 4, 42–46^, but never both TSR mechanisms acting together simultaneously against this herbicide, except in *Chloris truncata* ^47^. Ngo *et al*.^47^ suggested that the Glu-91-Ala mutation (position 86 in *Escherichia coli*) in a conserved region of the EPSPS gene contributed to the glyphosate resistance in *C. truncata*. However, the highly conserved region of the EPSPS gene was comprised of amino acid 90 to 102 in *E. coli* (from 95 to 107 in plants)^48^. Furthermore, Ngo *et al*.^47^ pointed to widely known mutations, induced in *E. coli* and plants, which may contribute to the glyphosate resistance, and they indicated that the Glu-91-Ala mutation had not been previously described as conferring resistance to this herbicide. Consequently, we suggest that the only TSR mechanism in *C. truncata* was the amplification of EPSPS. Therefore, in the case of the glyphosate resistance reported here, this is the first case of a weed involving both a mutation responsible for conferring resistance (Pro-106-Ser) as well as the overexpression of the EPSPS gene.

Goosegrass presents a great adaptive capacity to develop herbicide resistance, making it one of the most harmful weeds in agriculture; this weed was the first to achieve the following: evolved single Pro-106^1^ and double Thr-102-Ile + Pro-106-Ser^4, 16^ mutations that confer resistance to glyphosate; exhibited multiple resistance to non-selective herbicides^8^, and the current methodologies do not allow determination of the NTRS and TSR mechanisms involved in their resistance to glufosinate^49^; and now we demonstrated that goosegrass can be expressed by two mechanisms simultaneously at the target site level against glyphosate.

## Conclusion

Reduced translocation is one mechanism of resistance to glyphosate that has evolved in resistant goosegrass biotypes from Mexico. Greater selection pressure exerted by glyphosate triggered a mutation at the Pro-106 position + overexpression of the EPSPS gene in the resistant goosegrass biotypes in Persian lime groves. These results confirm the first case of glyphosate resistance of this species in the country, as well as the third case of resistance to this herbicide in Mexico’s citrus groves.

## Materials and Methods

### Plant Material

Within a citrus fruit region in the municipality of Martinez de la Torre, in the state of Veracruz, Mexico, seeds of fully mature goosegrass plants of two suspect R biotypes (Ein-Pl1: N 20°10′34, W 97°07′36; and Ein-Pl2; N 20°10′32, W 97°07′37) were collected in Persian lime groves and one (Ein-Or: N 20°10′28, W 97°07′12) from an orange grove. A susceptible biotype (Ein-S: N 20°10′17, W 97°06′47) without history of glyphosate application also was also collected in a land urban as control. The citrus fruit groves had a history of glyphosate usage of over 15 years with 3–4 and 1–2 applications per year of 720 g per ha in Persian lime and orange, respectively.

The seeds were germinated in peat, and the seedlings were transplanted individually into 250 mL pots with a substrate mixture of sand and peat (1: 1, v/v). The plants were placed in a greenhouse at a temperature regime of 26/18 °C day/night, and were treated with three-four true leaves counted from the bottom.

### Dose response assay

Different doses of glyphosate (Roundup EnergyPro 45% p/v [potassium salt]; Monsanto, Spain) were made with a Generation III Research Track Sprayer (DeVries Manufacturing Inc., Minnesota, USA) equipped with a flat-fan nozzle (Tee Jet 8002EVS) delivering 200 L ha^−1^ at 200 kPa. The glyphosate doses applied were: 0, 45, 90, 180, 360, 720, 1080, 2160, and 4320 g per ha. The experiments were arranged in a completely random design with 10 replications per dose and repeated twice. Survival and fresh weights of plants were measured at 21 DAT. Data were expressed as percent with respect to untreated control plants.

### Shikimic assay

Young leaf discs 4 mm in diameter were taken until completing 50 mg of plant tissue from plants of the goosegrass biotypes. Shikimic acid accumulation was determined according to Shaner *et al*.^50^. The glyphosate concentrations used were: 0, 0.1, 1, 10, 50, 100, 200, 400, 600 and 1000 µM. Sample absorbance was measured in a Beckman DU-640 spectrophotometer at 380 nm. The test was performed in triplicate on five treated and non-treated plants of each biotype in a completely random design. Results were expressed in mg of shikimic acid g^−1^ fresh tissue.

### ^14^C-glyphosate absorption and translocation

^14^C-glyphosate (American Radiolabeled Chemicals, Inc., USA) was added to the commercial herbicide to prepare a solution with a specific activity of 0.834 kBq μL^−1^. The final glyphosate concentration corresponded to 360 g ae per ha in 200 L ha^−1^. Goosegrass plants were treated (0.834 kBq plant^−1^) at 12, 24, 48, 72 and 96 HAT. Five plants per biotype at each time evaluated in a completely random design were handled according to Bracamonte *et al*.^38^. Radioactivity was analyzed by liquid scintillation spectrometry (LSS) in a Beckman LS 6500 scintillation counter (Beckman Coulter Inc. Fullerton, USA) during 10 min per sample. The average of ^14^C recovered (SE) was 93.6 (4.3) %. Percentage of ^14^C-glyphosate absorbed was expressed as [kBq in combusted tissue/(kBq in combusted tissue + kBq in leaf washes)] × 100.

### Metabolism

Six plants of each biotype were treated with 360 g ae per ha of glyphosate (as described in the dose-response assays) in a completely randomized design. Untreated plants were used as controls. Leaf tissues were washed with distilled water at 4 DAT, flash-frozen in liquid nitrogen, and stored at −40 °C until use. Following the methodology described by Rojano-Delgado *et al*.^51^, glyphosate and its metabolites (aminomethyl phosphonate (AMPA), glyoxylate, sarcosine and formaldehyde) were determined by reversed polarity capillary electrophoresis using a 3D Capillary Electrophoresis Agilent G1600A instrument equipped with a diode array detector (DAD, wavelength range 190–600 nm). Standard compounds used (glyphosate, AMPA, sarcosine, formaldehyde, and glyoxylate), were provided by Sigma-Aldrich, Spain. Glyoxylate naturally produced (untreated plants) was subtracted from the average of glyoxylate produced from glyphosate metabolism (treated plants) for each biotype.

### EPSPS enzyme assays

Five g of the leaf tissue of each biotype plants was ground to fine powder in liquid nitrogen in a chilled mortar. The enzyme extraction was conducted according to the protocol described by Sammons *et al*.^52^. The total soluble protein (TPS) in the extract was measured using a Modified Lowry Kit for Protein Determination (Sigma-Aldrich, Madrid, Spain) in accordance with the manufacturer’s instructions. The specific EPSPS activity was determined using the EnzCheck Phosphate Assay Kit (Invitrogen, Carlsbad, CA) following the manufacturer’s instructions, to determine the inorganic phosphate release. The glyphosate concentrations used were: 0, 0.1, 1, 10, 100, 200 and 1000 µM. The EPSPS activity was measured during 10 minutes at 360 nm in a spectrophotometer (Beckman DU-640) to determine the amount of phosphate (µmol) released µg of TSP^−1^ min^−1^ and expressed as a percentage with respect to the control (without glyphosate). The experiment was repeated three times for all samples.

### EPSPS gene sequence

Samples of young leaf tissue (≈100 mg) from 10 individual plants within each goosegrass biotype were ground to a fine powder in liquid nitrogen. Genomic DNA (gDNA) was extracted using Speedtools plant DNA extraction kit (Biotools, Madrid, Spain). Integrity of DNA was verified in 0.8% agarose gel and quantified in a NanoDrop ND-1000 spectrophotometer (Thermo Scientific, Walthman, MA, USA), and diluted to a final concentration of 50 ng 1 µL^−1^ and at −20 °C until use. The primers (forward: 5′-GGTGGATAACCTTTTAAACAGTGAG-3′; and reverse: 5′-TTAGTTCTTGACGAAAGTGCTGAGC-3′) designed by Chen *et al*.^4^, were used to amplify a fragment of 1300 pb in length, and the PCR conditions described by them were followed. An aliquot of the PCR product was loaded in a 1% agarose gel to check the correct band amplification. Then, the PCR product was purified using ExoSAP-IT® for PCR Product Clean-Up (USB, Ohio, USA) as indicated by the manufacturer. Fifteen µL of purified PCR product per sample was sequenced (STAB VIDA, Caparica, Portugal). Each individual was sequenced in triplicate. The resulting fragments were aligned with known EPSPS sequences of goosegrass from the GenBank.

### EPSPS gene expression

Six untreated plants with 5–6 true leaves of each biotype were sampled (one expanded leaf) before glyphosate treatment. Then, plants were treated with 360 g ae per ha of glyphosate (as previously described in the dose-response assays), and plant were sampled again at 24 and 48 HAT. Samples were immediately stored at −80 °C. RNA was extracted using TRIzol reagent (Invitrogen, Carlsbad, CA, USA) according to the manufacturer’s instructions. RNA was then treated with TURBO DNase (RNase-Free; Ambion, Warrington, UK) to eliminate any DNA contamination. Integrity of RNA was verified in 0.8% agarose gel and quantified in a NanoDrop ND-1000. First strand complementary DNA (cDNA) synthesis was carried out using one µg of RNA in all the samples. An iScript cDNA Synthesis Kit (Bio-Rad Laboratories, Inc. CA, USA) was employed following the manufacturer’s instructions. For qPCR analyses, the EPSPS (GF: 5′-CTGATGGCTGCTCCTTTAGCTC-3′; and GR: 5′-CCCAGCTATCAGAATGCTCTGC-3′), and *β*-Actin (F1a: 5′-ATGGTAGGGATGGGACAGAA-3′; and R1a: 5′-TCCATGTCATCCCAGTTGCT-3′) primers designed by Chen *et al*.^4^ and Alcántara-de la Cruz *et al*.^18^, respectively were used. The PCR reactions were carried out using an ABI Prism 7500 sequence detection system (Applied Biosystems, USA). Quantitative RT-PCR conditions and EPSPS gene expression analyzes were performed according to Alcántara-de la Cruz *et al*.^18^.

EPSPS expression level was calculated for each qPCR reaction. The PCR efficiency of each biotype sample and the stability of the *β*-Actin, as a reference gene, were determined using geNorm software according to Vandesompele *et al*.^53^. Three-four technical replications per plant were analyzed in a randomized design.

### Statistical analysis

Dose-response and EPSPS enzyme activity data were subjected to non-linear regression analysis using a log-logistic equation: *Y* = *c* + {(*d* − *c*)/[1 + (*x*/*g*)^*b*^]}; where *Y* is the percentage of fresh weight, survival and/or EPSPS-inhibiting with respect to the control, *c* and *d* are the parameters corresponding to the lower and upper asymptotes, *b* is the slope of the curve at the inflection point, *g* the herbicide rate at the inflection point (i.e. GR_50_, LD_50_ or I_50_), and *x* (independent variable) is the glyphosate dose; to find out the amount of glyphosate needed to reduce the fresh weight (GR_50_), mortality (LD_50_), and to inhibit EPSPS activity (I_50_) by 50% of each goosegrass biotype. Regression analyses were conducted using the *drc* package with program R version 3.2.5^54^, and the data were plotted using SigmaPlot 11.0 (Systat Software, Inc., USA).

Data of absorption and translocation, metabolism and EPSPS expression were subjected to ANOVA using Statistix version 9.0 from Analytical Software (Tallahassee, FL, USA). Model assumptions of normal distribution of errors and homogeneous variance were graphically inspected. When necessary, the means were compared using Tukey’s test’s at the 95% probability level.
